# Production of a cellular product consisting of monocytes stimulated with Sylatron^®^ (Peginterferon alfa-2b) and Actimmune^®^ (Interferon gamma-1b) for human use

**DOI:** 10.1186/s12967-019-1822-6

**Published:** 2019-03-14

**Authors:** Daniel S. Green, Ana T. Nunes, Kevin W. Tosh, Virginia David-Ocampo, Vicki S. Fellowes, Jiaqiang Ren, Jianjian Jin, Sue-Ellen Frodigh, Chauha Pham, Jolynn Procter, Celina Tran, Irene Ekwede, Hanh Khuu, David F. Stroncek, Steven L. Highfill, Kathryn C. Zoon, Christina M. Annunziata

**Affiliations:** 1 Women’s Malignancy Branch, Center for Cancer Research, National Cancer Institute, National Institutes of Health, 10 Center Drive RM 3B43C, Bethesda, MD 20892 USA; 2 Cell Processing Section, Department of Transfusion Medicine, Clinical Center, National Institutes of Health, Bethesda, MD USA; 3 Laboratory of Infectious Diseases, National Institutes of Allergy and Infectious Diseases, National Institutes of Health, Bethesda, MD USA; 4 Laboratory of Parasitic Diseases, National Institutes of Allergy and Infectious Diseases, National Institutes of Health, Bethesda, MD USA; 5 Present Address: Office of Tissues and Advanced Therapies, Center for Biologics and Evaluation and Research, FDA, Silver Spring, MD USA

**Keywords:** Cell therapy, Cellular immunotherapy, Monocytes, Interferons, Innate immunity

## Abstract

**Background:**

Monocytes are myeloid cells that reside in the blood and bone marrow and respond to inflammation. At the site of inflammation, monocytes express cytokines and chemokines. Monocytes have been shown to be cytotoxic to tumor cells in the presence of pro-inflammatory cytokines such as Interferon Alpha, Interferon Gamma, and IL-6. We have previously shown that monocytes stimulated with both interferons (IFNs) results in synergistic killing of ovarian cancer cells. We translated these observations to an ongoing clinical trial using adoptive cell transfer of autologous monocytes stimulated ex vivo with IFNs and infused into the peritoneal cavity of patients with advanced, chemotherapy resistant, ovarian cancer. Here we describe the optimization of the monocyte elutriation protocol and a cryopreservation protocol of the monocytes isolated from peripheral blood.

**Methods:**

Counter flow elutriation was performed on healthy donors or women with ovarian cancer. The monocyte-containing, RO-fraction was assessed for total monocyte number, purity, viability, and cytotoxicity with and without a cryopreservation step. All five fractions obtained from the elutriation procedure were also assessed by flow cytometry to measure the percent of immune cell subsets in each fraction.

**Results:**

Both iterative monocyte isolation using counter flow elutriation or cryopreservation following counter flow elutriation can yield over 2 billion monocytes for each donor with high purity. We also show that the monocytes are stable, viable, and retain cytotoxic functions when cultured with IFNs.

**Conclusion:**

Large scale isolation of monocytes from both healthy donors and patients with advanced, chemotherapy resistant ovarian cancer, can be achieved with high total number of monocytes. These monocytes can be cryopreserved and maintain viability and cytotoxic function. All of the elutriated cell fractions contain ample immune cells which could be used for other cell therapy-based applications.

**Electronic supplementary material:**

The online version of this article (10.1186/s12967-019-1822-6) contains supplementary material, which is available to authorized users.

## Background

Adoptive cell therapy (ACT) for the treatment of cancer was pioneered in the 1980s using T cells harvested from the patients’ own tumors [1]. Since then, autologous cellular immunotherapy approaches have expanded from using endogenous TILs to engineering cells to express selected T cell receptors [2] or to express chimeric antigen receptors that are not restricted by HLA type [3]. The CAR approach has been reproducibly successful in targeting CD19 in B cell acute lymphoblastic leukemia (ALL), leading to the first Federal Drug Administration (FDA) approval of Tisagenlecleucel in 2017. Shortly thereafter, the FDA approved Axicabtagene ciloleucel for the treatment of diffuse large B cell lymphoma.

ACT is derived from the observations that immune cells recognize and kill cancer cells [4]. Based on these observations it was posed that the anti-inflammatory environment of the tumor inhibited a de novo immune response. Clinical trials have tested numerous strategies for re-activating lymphocytes and other leukocyte subsets [5]. We chose a complementary approach, focusing on innate immunity [6]. The innate immune system, including monocytes, macrophages and NK cells, also plays a crucial function in controlling cancer [7]. Our initial studies re-examined the innate immune system as anti-cancer therapy. We showed that IFNα-2a or IFNγ-1b themselves are potently anti-neoplastic in vitro and in mouse models of ovarian cancer, and the effect was amplified with the addition of monocytes [8].

Activated monocytes are capable of killing malignant cells [9]. Within tissues, monocytes can differentiate into inflammatory M1 macrophages with anti-cancer activity or suppressive M2 macrophages that promote tumor proliferation [10–12]. M2 macrophages are associated with poor prognosis in advanced epithelial ovarian cancer [13]. Therefore, the success of monocytes as an anti-tumor treatment approach depends on the ability to maintain M1 phenotype and avoid M2 differentiation in the tumor micro-environment. Importantly, our previous work showed both in vitro and in animal models, monocytes differentiated into M1 macrophages the presence of IFNα and IFNγ (increased IL-12, CXCL10, NOS2, and decreased IL-10, Arginase-1) [8]. We previously showed that monocytes stimulated with both IFNs are cytotoxic to six different ovarian cancer cell lines, and that this combination significantly improved tumor cell response to carboplatin and paclitaxel in vitro [14]. In mouse xenografts, intratumoral injection of monocytes with IFNs decreased ovarian cancer xenograft growth. With these promising results, we were encouraged to take this combination therapy forward to the clinical setting, while continuing to explore the molecular mechanism underlying the synergy between monocytes and interferons [15]. We designed a clinical trial to test the safety of four different dose combinations of monocytes and IFNs (Table 1).

**Table 1:** Final product packaging and stability testing

	Volume (mL)	viable cell concentration	Viable total nucleated cells	% TB viability	% Viable monocytes
Donor 1: 0-h	250	3.30E+05	8.25E+07	97	81.2
2-h stability (RT)	250	3.20E+05	8.00E+07	99	NA
4-h stability (RT)	250	3.40E+05	8.50E+07	97	80.3
Donor 2: 0-h	250	3.25E+05	8.13E+07	100	76.9
2-h stability (RT)	250	3.40E+05	8.50E+07	98	NA
4-h stability (RT)	250	3.05E+05	7.63E+07	97	64.9
Donor 3: 0-h	250	4.68E+06	1.17E+09	98	66
2-h stability (RT)	250	4.89E+06	1.22E+09	96	NA
4-h stability (RT)	250	4.47E+06	1.12E+09	93	66.9
Donor 4: 0-h	250	4.08E+06	1.02E+09	94	74.2
2-h stability (RT)	250	3.53E+06	8.83E+08	96	NA
4-h stability (RT)	250	3.31E+06	8.28E+08	95	73.7

Methods for collecting monocytes have been optimized for the purpose of preparing dendritic cell vaccines [16]. In this procedure, the RO fraction contains the greatest number of monocytes. Despite the long historical use of counter flow elutriation, however, an analysis of critical immune cells in all 5 fractions has not been performed. Here, we define the sub-populations of immune cells in each fraction, show reproducibility of the product in producing large numbers of viable monocytes, and create a cryopreservation step to store multiple doses of the product from a single leukapheresis procedure.

## Methods

### Primary cell toxicity

Primary hepatocytes were purchased from Life Tech, pre-plated on 96-well plates. All hepatocyte donors were female (n = 4) All other primary cells were grown under culture conditions with specific media per manufacturer’s instructions (Lonza). The sex of the donors for these cells is unknown. All cells were assayed on passage 1, 2 and 3. Three healthy donor elutriated monocytes were used for each experiment (all female). Monocytes purity were from the DTM at 87%, 89%, and 90% purity. Cells were assayed in the standard monocyte assay. Briefly monocytes were incubated with indicated cells with and without Sylatron^®^ (Peginterferon alfa-2b) and Actimmune^®^ (Interferon gamma-1b) for 72-h incubation. Cells measured for viability using crystal violet assay as this is the most definitive assay for measuring cell viability due to the ability to directly assess the cancer cells. We chose not to use Annexin V assays because the myeloid cells in the assay express high amounts of phosphatidyl serine on their outer membrane when alive, and their binding of Annexin V would confound the results. Interferon concentrations were escalated within clinically achievable range. Staurosporine was used as a positive control of cell death.

**Fig. 1:**
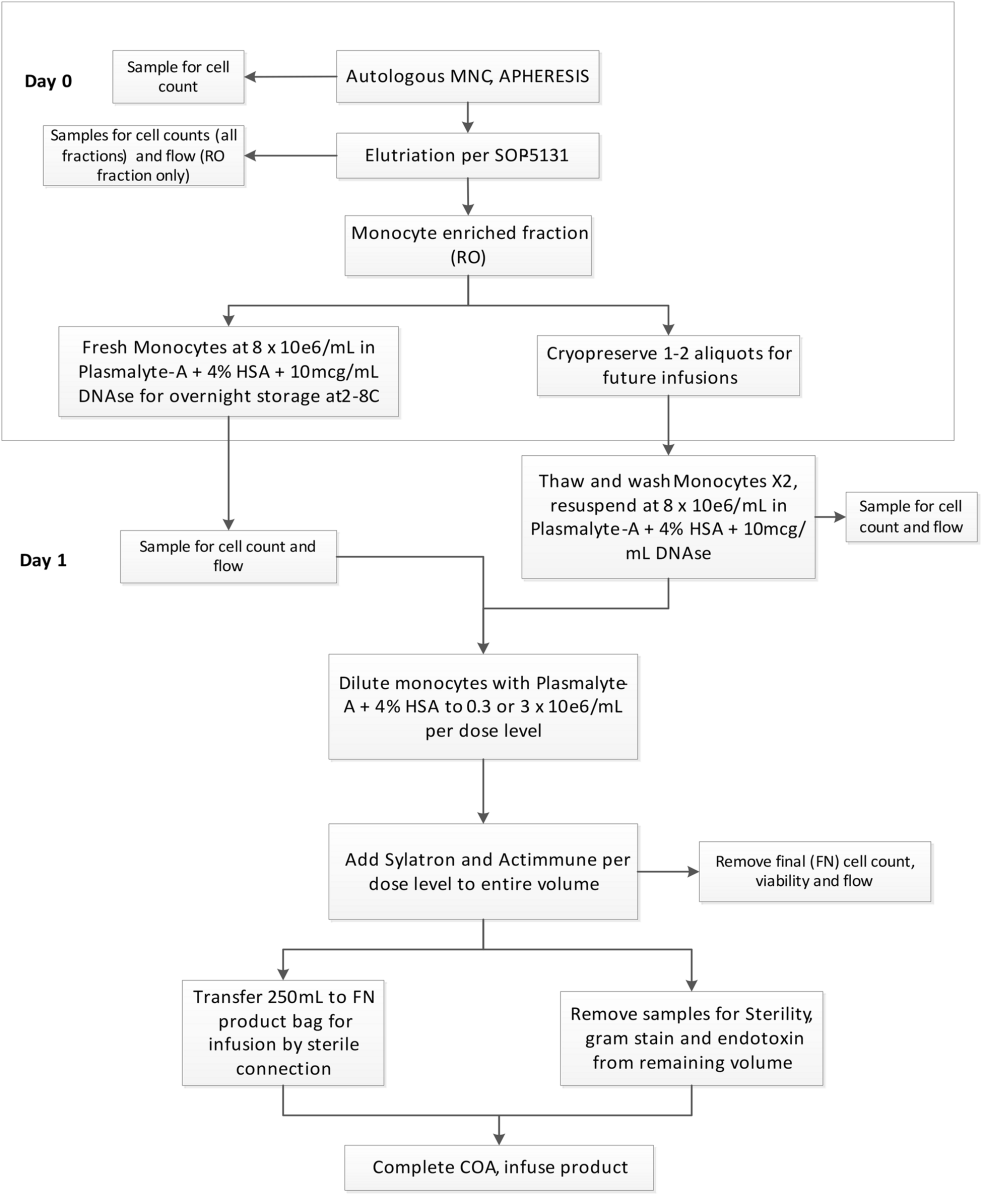
Work flow of product creation from for fresh and frozen monocytes. This schematic shows the work flow from apheresis to either creation of the final product using fresh or cryopreserved monocytes

Patients underwent a 7–15 L PBMNC apheresis (autologous), as estimated by weight and target cell dose. Bilateral peripheral venous access was used whenever possible. Alternatively, a temporary central venous catheter (CVL) was placed for collection. Cells were processed for further manufacturing (Fig. 1).

Cells manufactured in the current protocol are of autologous nature and donor eligibility determinations are not required (21 CFR 1271.90(a)(1)) [17]. However, eligibility requirements for product handling in our manufacturing facility require that subjects be HIV seronegative, hepatitis C seronegative and negative for Hepatitis B surface antigen. In addition, on the day of product collection, peripheral blood is submitted for testing: HIV 1 and 2 (antibody and NAT), HTLV I and II (antibody), Hepatitis C (antibody and NAT), Hepatitis B (surface antigen), West Nile Virus (NAT), T cruzi (antibody), and Treponema pallidum (antibody).

No feeder cells or cell lines were used in this protocol.

### Fresh product

Apheresis products from four normal volunteer donors were collected sequentially in DTM within 2 weeks of one another. On the day of collection (day 0), the apheresis product was enriched for monocytes using counter-flow centrifugation (CCE) [18]. The enriched monocyte fraction was evaluated for total cell number and purity using the CellDyn automated cell counter. The product was then diluted with Plasmalyte A/4% HSA and 10 mcg/mL DNAse (Pulmozyme) for overnight storage. On the next day (day 1), the final product was prepared with Peginterferon alpha 2b (*Sylatron™*) and Interferon gamma-1b (*Actimmune*^*®*^). Saturating levels of IFNs are added to the final product. For example, monocytes express approximately 5 × 10^3^ receptors/cell. With 5 × 10^5^ cells/mL, there are 2.5 × 10^9^ receptors to fill. A concentration of 200 ng/mL IFNa is approximately 10 pmol, which is 6 × 10^12^ molecules, giving an excess of 2.4 × 10^3^ molecules per receptor.

### Cryopreserved product

We designed and validated a new process that would allow us to prepare one initial fresh dose of monocytes and cryopreserve the remaining cells so that they may be thawed and infused at a later date. This new modification would reduce stress to the patient due to having to undergo multiple apheresis and relieves the burden of performing the procedure in a single apheresis clinic. Three separate samples were tested independently, two samples from healthy donors and one sample taken from a patient with ovarian cancer. Each sample was processed as a fresh and as a cryopreserved specimen.

Elutriated monocytes were cryopreserved with freeze mix containing 5% dimethyl sulfoxide (DMSO) as an intracellular cryoprotectant and 6% pentastarch as an extracellular cryoprotectant and 3.75% human serum albumin (HSA). Heparin and dornase alfa (DNAse) were added to minimize clumping at the time of thaw. Cells combined with freeze mix were cryopreserved in a controlled rate freezer (CRF) to a temperature of − 110 to − 120 °C and then stored in vapor phase of a liquid nitrogen (LN2) tank at − 150 to − 180 °C. Cells from donors were cryopreserved in aliquots 330 × 10^6^ TNC (260 × 10^6^ CD14/CD16 monocytes) and 990 × 10^6^ TNC (780 × 10^6^ CD14/CD16 monocytes).

### Reagent information for creation of the final product

All product names, national drug code, and regulatory status can be found in Additional file 1: Table S14.

### Statistical analysis

Comparisons were made using two-way ANOVA with Tukey post hoc analysis to correct for multiple comparisons.

## Results

### Iterative isolation and production of fresh monocytes

**Fig. 2:**
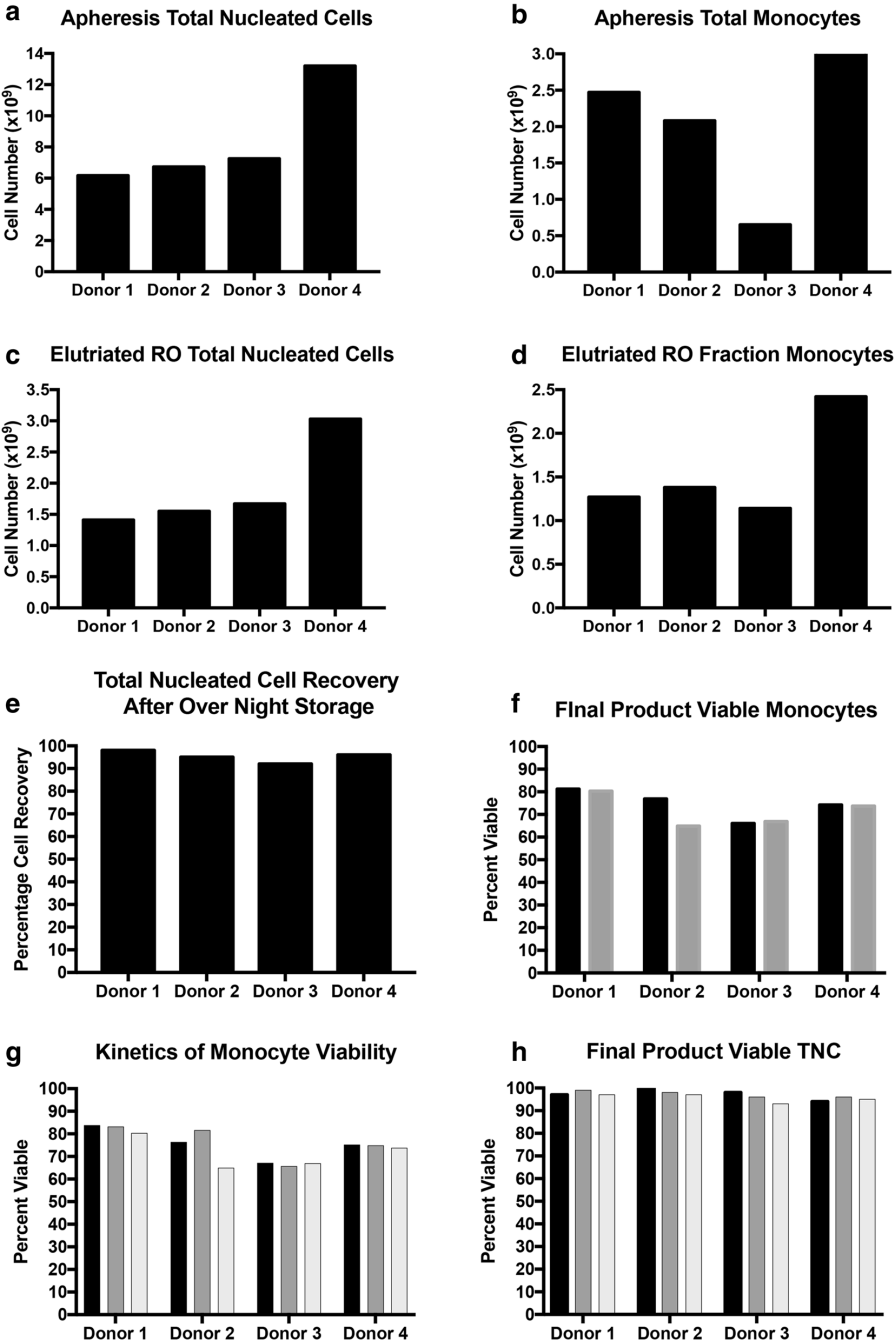
Quantification of cell numbers and viability. Apheresis followed by counter-flow elutriation was performed on 4 healthy donors.** a** Total nucleated cells and total monocytes from the bullk apheresis shown for all donors as total cell number. **b** Total monocytes from the bulk apheresis. **c** Total Nucleated Cells in the RO fraction. **d** Total monocytes in the RO fraction. **e** Recovery of total nucleated cells from overnight storage was measured. **f** Viability of the final product monocytes at 0 h (black bars) and 4 h (grey bars) was measured. **g** Kinetics monocyte viability the on day 1 (black bars), day 2 (grey bars) and the final product were measured. **h** Total nucleated cell count was performed on the final product at 0 h (black bars), 2 h (grey bars), and 4 h (white bars)

**Fig. 3:**
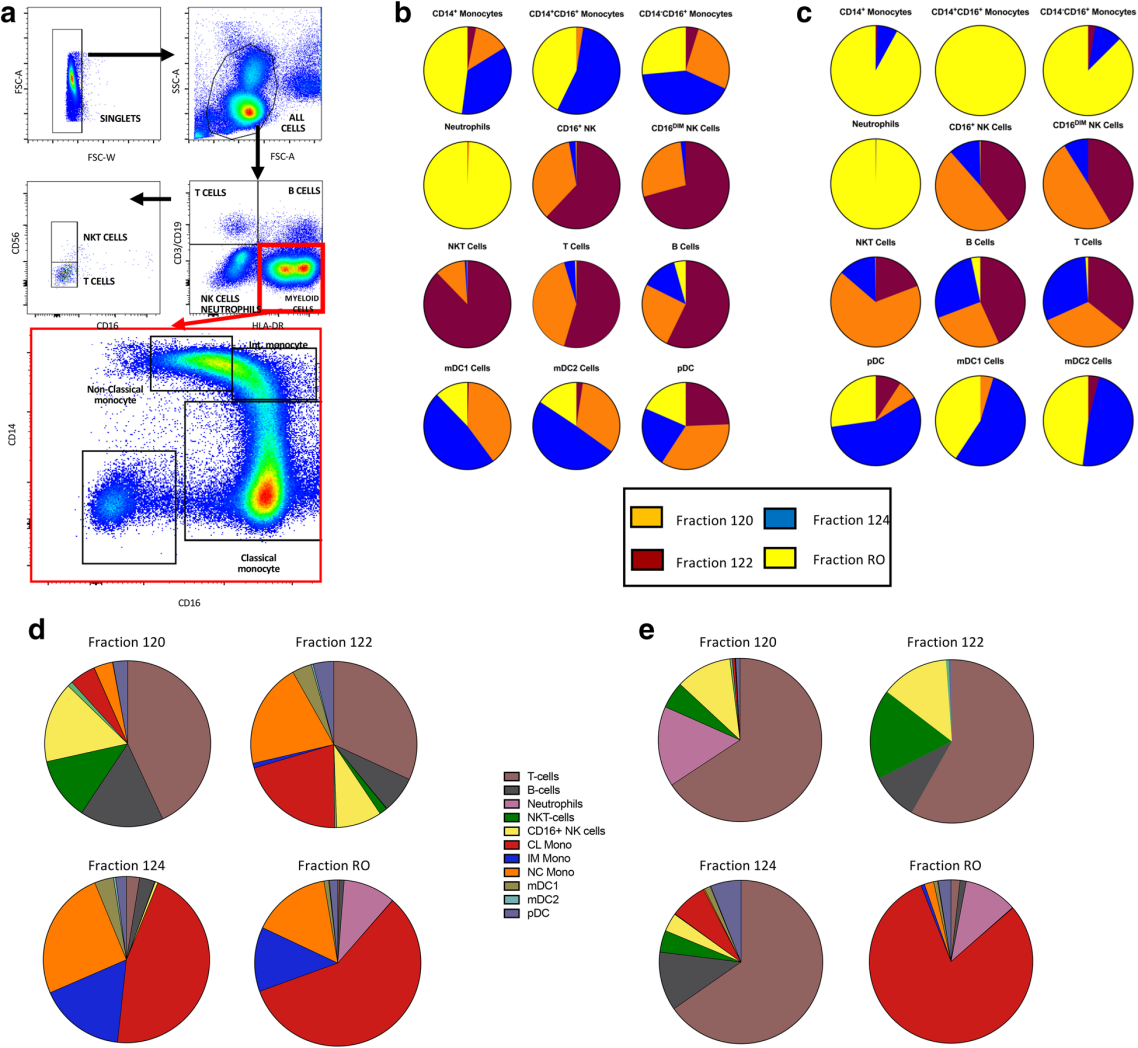
Flow cytometric analysis and quantification of immune subtypes in the four fractions of counter-flow elutriation. **a** A condensed work flow of flow cytometry of of cells from counter flow elutriation, with a focus on the monocyte population (red box). **b** Percentages of immune subtypes in all fractions from healthy donor 1. **c** Percentages of immune subtypes in all fractions from healthy donor 2. **d** Proportion of each immune subtype by fraction of elutriation for healthy donor 1. **e** Proportion of immune subtypes by fraction of elutriation for healthy donor 2 (complete work-flow analysis in Additional file 1: Table S3, Additional file 2: Figure S1, Additional file 3: Figure S2, Additional file 4: Figure S3 and Additional file 5: Figure S4)

Blood from healthy donors was used to validate the process. Seven to 8 L blood apheresis was collected from volunteer donors with TNC range of 6.17 × 10^9^–13.2 × 10^9^ and total monocytes of 6.52 × 10^8^–3.29 × 10^9^ on automated cell counter CellDyn (Fig. 2a and Additional file 1: Table S1). Elutriation was performed on the same day of collection to enrich for monocytes and sample removed from each fraction post volume reduction to evaluate recovery and purity. Monocytes recovered from elutriated RO fraction from all four donors were sufficient to prepare final product at each dose level (Table 1). We quantified immune subtypes by flow cytometric analysis of the four fractions of counter-flow elutriation, defining T cells (CD3), B cells (CD19), myeloid cells (HLA-DR), and NK cells/neutrophils (negative) (Fig. 3a). Monocytes were further segregated from the myeloid population by CD14^+^CD16^−^ (classical), CD14^+^CD16^+^ (Intermediate) and CD14^−^CD16^+^ (non-classical). Monocytes from healthy donor #1 were predominantly in the RO fraction, along with neutrophils and some of the dendritic cells (Fig. 3b, d). In donor #2, monocytes were found in the RO fraction, and also in fraction 124, which also contained a large fraction of the dendritic cells (Fig. 3c, e). Complete work-flow analysis is shown in Additional file 1: Table S2, Additional file 2: Figure S1, Additional file 3: Figure S2, Additional file 4: Figure S3 and Additional file 5: Figure S4.

Monocytes from RO fraction was transferred into a 600 mL Terumo bag and spun down to remove residual HBSS from elutriation procedure. Cells were then re-suspended in solution containing Plasmalyte-A/4% HSA/10 mcg/mL DNAse (Pulmozyme) at cell concentration of 5–10 × 10^6^/mL. A sample was removed for automated count, Trypan Blue viability and FACs prior to storage per SOP. Cell viability and monocyte purity before overnight storage from all four donors averaged ≥ 97% and 75.5% respectively (Fig. 2b and Additional file 1: Table S2).

The following day (day 1), stored cells were removed from refrigerator to warm to room temperature and filtered through a blood component recipient set to remove cell aggregates. A sample was removed for an automated cell count, TB viability and flow to evaluate for viability and cell loss. Average cell viability post storage from all donors remained > 95% with cell recovery > 92% (Fig. 2b, Additional file 1: Tables S4, S5).

**Fig. 4:**
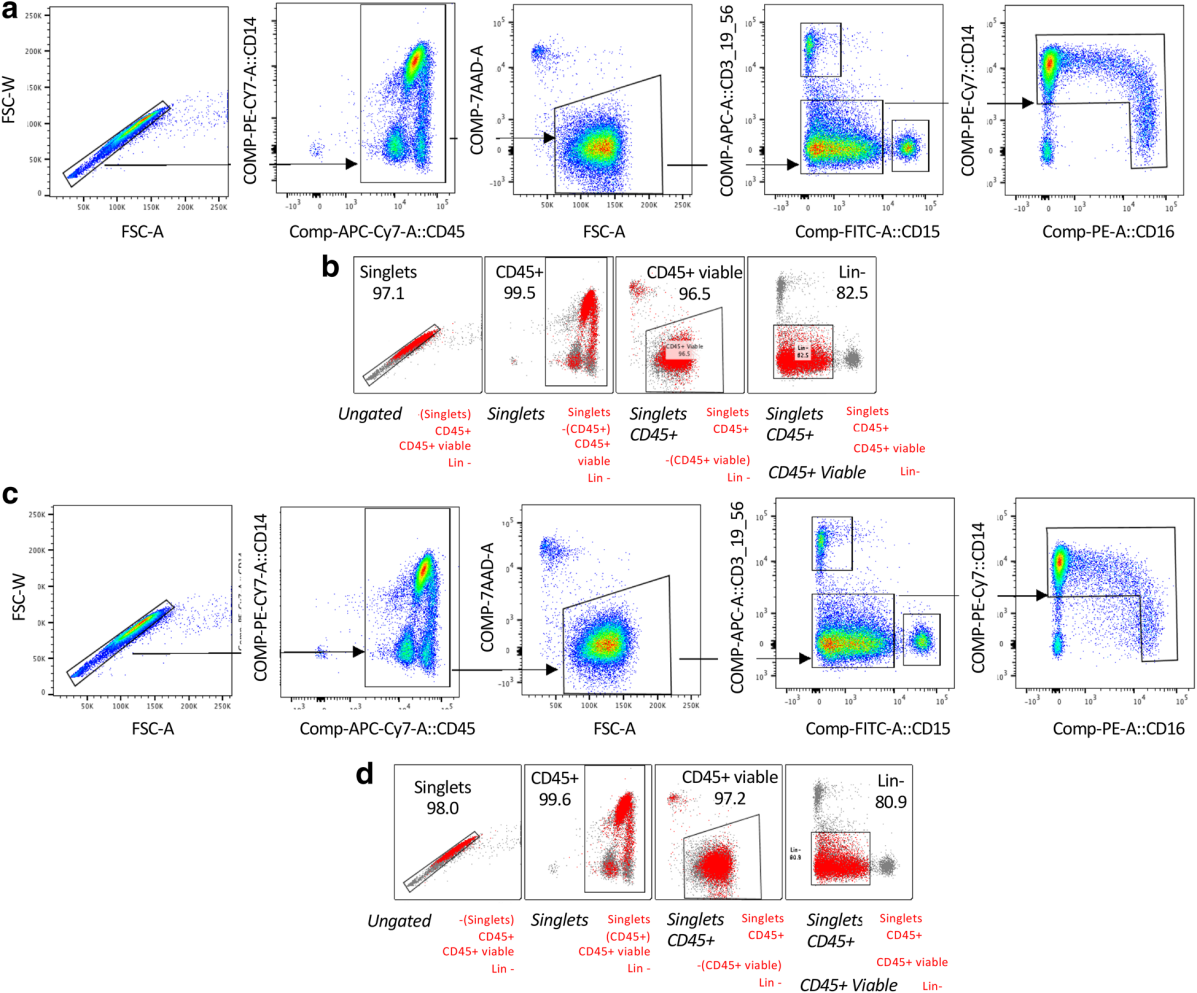
Workflow for elutriated fraction RO for the quantification of percent monocytes. **a** Cells from the final product from a healthy donor after the addition of interferons were gated on singlets, followed by CD45^+^ cells, live cells, and then the CD15^−^Lin^−^ population. Total monocytes were counted as the combination of Classical (CD14^+^), intermediate (CD14^+^CD16^+^), and non-classical CD14^−^/^dim^CD16^+^. **b** Shows the backgating of the monocyte population. **c** Analysis of the same product from **a** after 4 h incubation at room temperature. **d** Shows the backgating strategy as in **b**

The final product packaging on the first two donors was prepared at dose level 2 with 75 × 10^6^ viable monocytes, 25 mcg SylatronTM and 5 mcg Actimmune^®^. The last two donors were prepared at dose level 4 with 750 × 10^6^ viable monocytes with 250 mcg SylatronTM and 50 mcg Actimmune^®^. All final products were prepared in 250 mL of Plasmalyte-A and 4% HSA contained in a Terumo transfer bag. Automated cell count, % trypan blue (TB) viability, flow and safety testing were submitted. After the addition of IFNs, cells from the final product were gated on singlets, followed by CD45^+^ cells, live cells, and then the CD15^−^Lin^−^ population (Fig. 4). Total monocytes were counted as the combination of Classical (CD14^+^), intermediate (CD14^+^CD16^+^), and non-classical CD14^−/dim^CD16^+^ (Fig. 4a, b). All of these cells—classical, intermediate and non-classical—were included in the final product. Stability testing at room temperature (RT) for cell count and viability were performed 2 and 4 h post packaging. The viability at these time points remained > 90% for all four donors tested (Table 1). The monocyte population was analyzed after 4 h incubation at room temperature in order to confirm the stability of the population in the maximum time frame expected to complete initial sterility testing and transfer the product to the patient for intraperitoneal infusion (Fig. 4c, d).

Sterile cultures were submitted for bacterial/fungal sterility from the final product (FN). Results of Safety testing showed no growth (Additional file 1: Table S6). Mycoplasma PCR and Endotoxin testing was also negative.

### Storage of monocytes using cryopreservation

First, we examined cell recovery following cryopreservation to ensure the cells were capable of surviving the cryopreservation process. Cells from all three donors were cryopreserved in aliquots > 330 × 10^6^ TNC (or > 260 × 10^6^ CD14/CD16 monocytes) (Additional file 1: Table S7). These aliquots represent the typical cell number that would be cryopreserved for use on an interventional clinical trial under development. Upon thaw, cells were counted and recovery was calculated. Total nucleated cell (TNC) recovery from all three samples was > 88% (> 91% for CD14/CD16 monocytes). We conclude that there is no detrimental effect of the cryopreservation procedure on the TNC or monocyte recovery.

Next, we compared the frequency of CD14/CD16 cells that were in the initial apheresis, in the fresh elutriated sample, and in the cryopreserved sample. After elutriation, we observed a consistent increase in the frequency of monocytes that were within the RO fraction. The frequency ranged from 14 to 43% in the starting bag to > 75% post elutriation (Additional file 1: Table S8). There was < 5% difference when comparing the frequency of monocytes between stored monocytes (SM) and day 1 of fresh sample and cryopreserved sample. Similarly, there was < 9% difference when comparing the frequency of monocytes between final product (FN) samples of fresh compared to cryopreserved samples. Together, this data indicate that cryopreservation of the intermediate product did not significantly affect the monocyte frequency. Loss of CD14/CD16 monocytes that occurred during processing from day 1 until the FN was packaged was minimal. Fresh sample recovery during this time point was > 93% and cryopreserved sample recovery was > 86% (Additional file 1: Table S9).

Monocyte viability pre and post cryopreservation was examined next. Samples were taken at day 1 and in the final product after resuspension with cytokines and assessed for viability using AO/PI uptake on the Cellometer instrument. The viability of all samples > 92% (Table 2). In addition, we examined the stability of the monocytes post thaw at 2 h and 4 h time points after final packaging with cytokines to ensure that they remained stable while they are transferred to the clinic for administration to the patient. We found that the viability at these time points was not significantly different than the viability at the 0 h time point (all > 93%) and conclude that the monocytes would remain stable for up to 4 h (Table 3).

**Table 2:** Cell viability

Sample	Fresh sample		Cryo sample	
	Day 1 (%)	FN (%)	Day 1 (%)	FN (%)
1	99.0	98.0	99.6	98.9
2	97.0	94.5	92.8	93.8
3	99.0	99.5	96.4	98.8

**Table 3:** Stability of cryopreserved monocytes after thaw

Sample	FN, % (0 h)	FN, % (2 h)	FN, % (4 h)
1	98.9	98.6	99.0
2	93.8	94.8	93.6
3	98.8	98.3	98.2

**Fig. 5:**
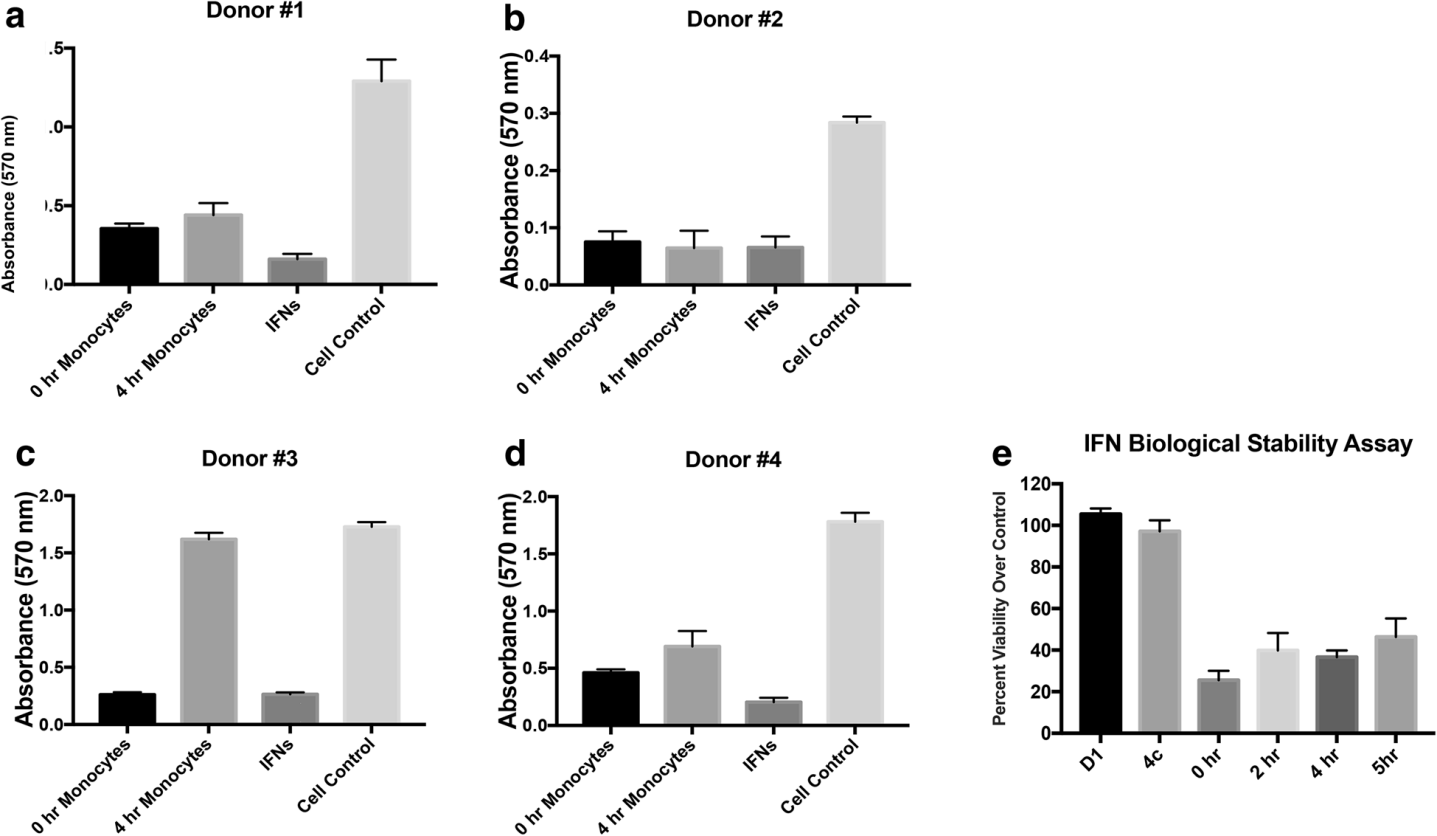
Cytotoxicity and IFN stability. Apheresis and counter-flow elutriation was performed on four donors. A final product was generated combining the monocytes with IFNs at the second dose level (**a**, **b**) and the fourth dose level (**c**, **d**). The products were assayed for cytotoxicity to OVCAR3 ovarian cancer cells. Time 0 h product (black bars), product stored 4 h at room temperature (light grey), IFNs alone (dark grey), and cell medium supernatant without IFNs (white bar) were measured. **e** IFN stability was measured after incubation at room temperature for 0, 2, 4, and 5 h; initial (D1) and overnight (4c) monocyte supernatants are used as controls, for cytotoxicity with OVCAR3 cells

**Fig. 6:**
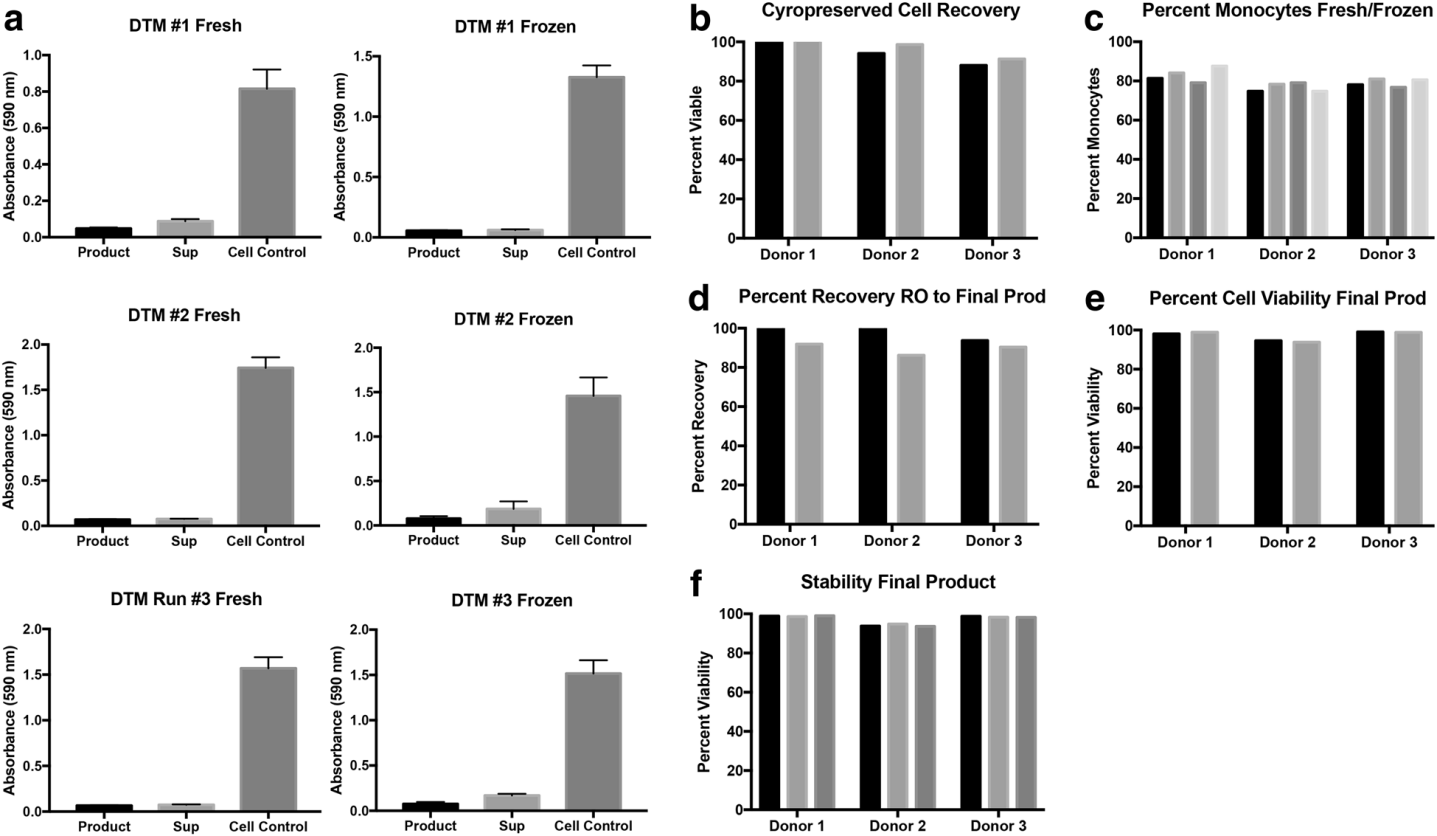
Cytotoxicity of the final product before and after cryopreservation. Three separate apheresis and elutriation runs were performed. **a** At the time of product release both final product (IFNs and monocytes) and the IFNs alone were assayed for toxicity using OVCAR3 cells as the target. **b** Percent viable total nucleated cells (black bars) and the final product (grey bars). **c** Percent fresh monocytes in the RO fraction (black bars), the RO cryopreserved (light grey bars), final product fresh (dark grey bars), and final product cryopreserved (white bars). **d** Percent recovery from the fresh RO fraction (black bars) and cryopreserved RO fraction (grey bars). **e** Percent viability of the fresh final product (black bars) and cryopreserved product (grey bars). **f** Stability of the product measured by monocyte viability at 0 h (black bars), 2 h (light grey bars), and 4 h (dark grey bars)

Functional assays were performed using the final product from both the fresh and cryopreserved samples. Monocytes plus IFNs were mixed with ovarian cancer cells in vitro and allowed to incubate together for 3 days. Monocyte and IFN cytotoxicity was measured as absorbance with cell control having maxima cell viability. The freshly prepared product remained effective in killing OVCAR3 ovarian cancer cells after 5 h at room temperature (Fig. 5). In addition, we tested cytotoxicity of the final product before and after cryopreservation. Three separate apheresis and elutriation runs were performed. At the time of product release, both final product (IFNs and monocytes) and the IFNs alone (supernatant) were assayed for toxicity using OVCAR3 cells as the target. Percent killing is calculated as (100% survival). There was no difference in cell killing effectiveness between the fresh and the frozen products or supernatants (Fig. 6). All samples were highly efficient in their ability to lyse target tumor cells. The percent killing from all samples was > 94%, and the difference of percent killing between individual fresh vs. cryopreserved samples was < 2% (Additional file 1: Table S10). These data indicate that no loss of function occurred within the cryopreserved samples. A product release check list was created for the final product (Table 4).

**Table 4:** Final release criteria

Test	Method	Acceptable limit
Appearance	Visual check	Normal–milky; no aggregates
% Viable monocytes (CD14+)	FACS	≥ 40%
Endotoxin	LAL assay	< 5EU/mL
Sterility on final product	Bacterial bottle cultures, fungal on plate cultures	No growth
Gram stain	Gram stain	No organism seen
Donor eligibility	NA	Auto

**Fig. 7:**
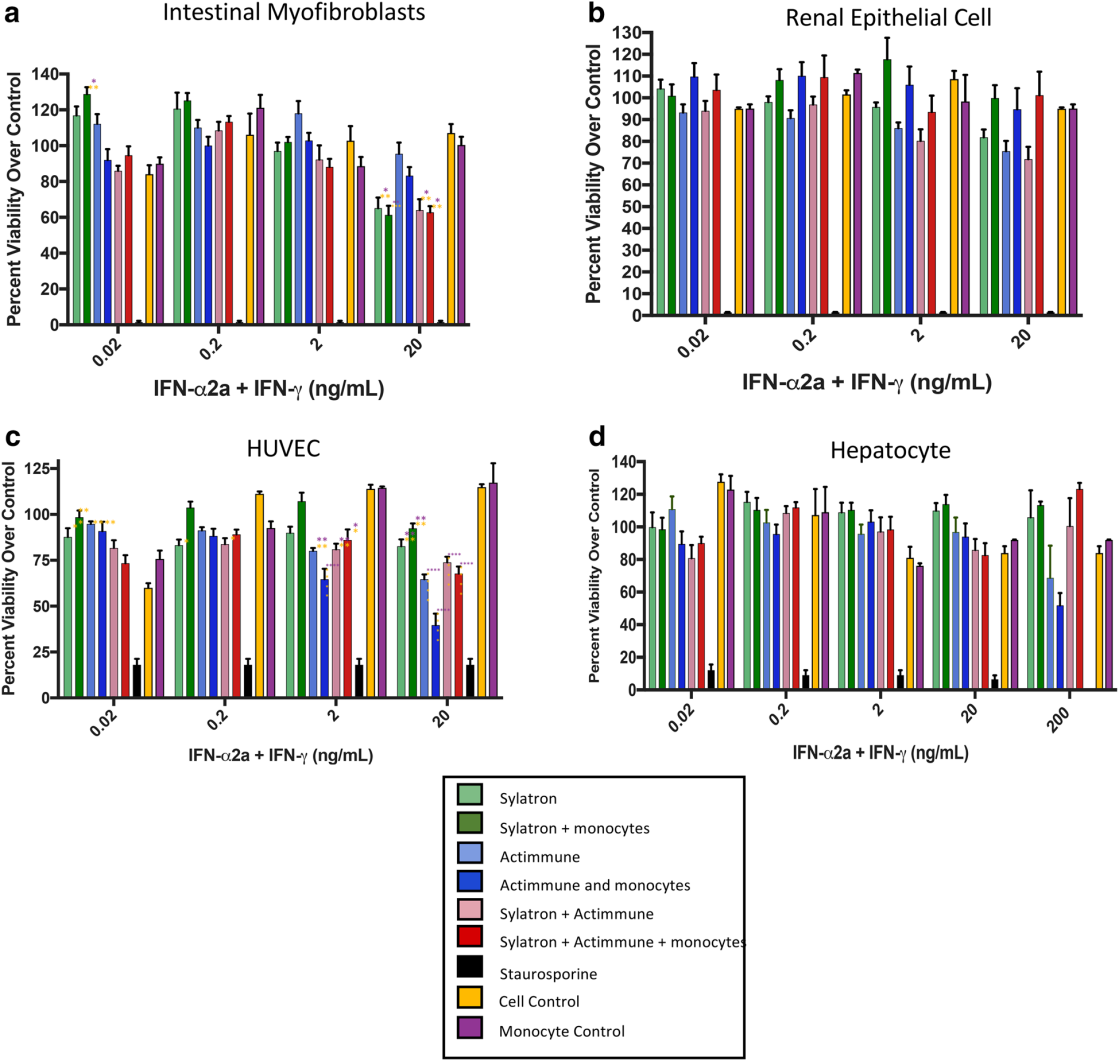
Toxicity screen of Sylatron and Actimmune on primary human cells. **a** Myofibroblasts, **b** renal epithelial cells, **c** human vascular endothelial cells (HUVEC), **d** primary hepatocytes were assayed with Sylatron (light green bars), Actimmune (light blue bars), Sylatron and Actimmune (light red bars) or Sylatron and monocytes (dark green bars), Actimmune and monocytes (dark blue bars), Sylatron, Actimmune, and monocytes (dark red bars) for 3 days and cell viability was measured using trypan blue. Staurosporine (black bars) was used as a positive control for cell toxicity. Experiments were performed three times with three healthy donor monocytes, except for the hepatocytes which were repeated three times with three separate primary hepatocyte donors. Yellow asterisks indicate comparison to cell control; purple indicate comparison to monocyte-only control. Numbers of asterisks indicate p-value as follows: * < 0.05; ** < 0.01; *** < 0.001; **** < 0.0001

In addition, we confirmed lack of toxicity to normal cells and tissues in vitro and in animals. Primary human hepatocytes, intestinal myofibroblasts, endothelial cells, and renal epithelial cells were purchased. Minimal decrease in viability occurred after 72 h exposure to IFNs alone and in combination with human monocytes in vitro, except in the endothelial cells at the highest concentrations of IFNs (Fig. 7). Five groups of six nude mice each were treated with vehicle or four different dose combinations of human IFNs and human monocytes, to mimic a dose-escalating clinical trial (Table 5). Necropsy was performed on all mice by a trained animal pathologist blinded to group allocation, and no definitive toxicity noted in any of the groups. One animal in the highest dose group had mild, multifocal, perivascular, lymphocytic infiltrate in the brain. Specific cause is unknown, but since this lesion was not seen in any other animal, the change was not thought to be treatment related. Mottled lungs were noted grossly at necropsy in several of the mice. This is a common incidental finding related to congestion/hemorrhage occurring at euthanasia. The lesion is often not present after tissues are processed. No biologically significant difference in the lungs was noted across the groups. Remaining lesions were sporadic, low in incidence and/or severity, and showed no relationship to dose or treatment.

**Table 5:** Mouse studies for IND application

Dose level	Monocytes total number	Sylatron^®^ Peginterferon alfa2b	Actimmune^®^ Interferon gamma-1b
Vehicle	0	0	0
Low dose	7.5 × 10^4^	17 ng	1.7 ng
Mid dose	7.5 × 10^5^	17 ng	1.7 ng
High dose	7.5 × 10^5^	100 ng	16.7 ng
Fivefold max	7.5 × 10^5^	500 ng	85 ng

## Discussion

We have designed a processing scheme for the development of a clinical product comprised of autologous monocytes activated with IFNs alpha and gamma. Unlike other cell therapies which require complex processing, this therapy can be performed in any hospital with a transfusion center. The procedures are reproducible and scalable to account for the escalating doses on our phase 1 clinical trial. In order to minimize impact on the patient, we integrated a storage procedure that maintained potency of the final product. Having established the preclinical safety data and validated the monocyte processing methods, we filed an Investigational New Drug (IND) application and are proceeding with the phase 1 clinical trial in women with recurrent ovarian cancer (NCT02948426).

The overall goal of this project is to optimize monocytes and IFNs as a therapeutic strategy for women with ovarian cancer, and to build on this backbone by introducing mechanistically synergistic agents in combination. While different combinations of IFNs and monocytes were tested in human clinical trials for their tumoricidal properties, the three have never been combined. Based on preclinical data showing increased efficacy of the triplet, we are taking a unique approach of stimulating innate immunity with intraperitoneal adoptive cell therapy, utilizing the strength of our institution’s cell processing facility to develop this clinically.

Immune profiling of each elutriation fraction, as shown in Fig. 3, demonstrates that there is donor heterogeneity. This observation is important because it indicates other cell populations that could be further purified for use in cell therapy. The immune profiling also highlights, in the context of monocyte isolation, that fractions 124 and RO may be pooled in order to maximize the number of monocytes for either fresh preparation or cryopreservation.

We hypothesize that autologous monocytes stimulated with IFNα and IFNγ will prime an immune response to control the intraperitoneal growth of ovarian cancer. Phase I studies of individual IFNs delivered IP defined an acceptable toxicity profile [19–25]. Key findings from these studies were tolerable side effect profile and prolonged intraperitoneal concentrations of IFNs. Two phase 1 trials studied IP infusion of autologous monocytes [26–28]. These studies also showed that IP administration of activated monocytes was safe and feasible in patients with peritoneal carcinomatosis. While these studies showed that IP administration of IFNα or IFNγ is a potential therapeutic treatment, no studies tested both IFNs together by themselves or with monocytes. Thus, it is important to optimize the administration of intraperitoneal adoptive cell therapy, confirm that the product infiltrates the tumor, and understand the mechanism of anti-cancer activity on which to build.

## Conclusions

We validated a new manufacturing process for the preparation of cryopreserved monocytes and found that the cell product was extremely consistent between cryopreserved fraction vs. fresh fraction and also consistent among the three different donors tested here. All samples tested passed the validation criteria set forth in the validation plan, which also included product safety testing (sterility, endotoxin). Our phase 1 clinical trial (NCT02948426) is ongoing and results will be reported after completion of enrollment.

## Additional files

**Additional file 1.** Additional tables.

**Additional file 2: Figure S1.** Expanded workflow of cells from counter flow elutriation showing flow cytometry results from RO fraction of Donor 1.

**Additional file 3: Figure S2.** Expanded workflow of cells from counter flow elutriation showing flow cytometry results from 124 fraction of Donor 1.

**Additional file 4: Figure S3.** Expanded workflow of cells from counter flow elutriation showing flow cytometry results from 122 fraction of Donor 1.

**Additional file 5: Figure S4.** Expanded workflow of cells from counter flow elutriation showing flow cytometry results from 120 fraction of Donor 1.
