# Supplementary material for: Production of a cellular product consisting of monocytes stimulated with Sylatron® (Peginterferon alfa-2b) and Actimmune® (Interferon gamma-1b) for human use
Source: J Transl Med. 2019 Mar 14;17:82. doi: 10.1186/s12967-019-1822-6 (PMC6419352; doi:10.1186/s12967-019-1822-6)
Supplement: Supplementary file 1 — Additional file 1. Additional tables. [file 12967_2019_1822_MOESM1_ESM.docx]

Additional Tables:

S1. Total Nucleated Cells (TNC) and total monocytes from apheresis bag and RO fraction enriched monocytes

| Donor # | Apheresis Bag | | Elutriated RO fraction | |
| --- | --- | --- | --- | --- |
|  | TNC | Total monocytes | TNC | Total monocytes |
| 1 | 6.17E+09 | 2.47E+09 | 1.41E+09 | 1.27E+09 |
| 2 | 6.72E+09 | 2.08E+09 | 1.55E+09 | 1.38E+09 |
| 3 | 7.25E+09 | 6.52E+08 | 1.67E+09 | 1.14E+09 |
| 4 | 1.32E+10 | 3.29E+09 | 3.03E+09 | 2.42E+09 |

S2. Percentages and total number of cells from the elutriation fraction from Figures 1B and 1C

S3. Supplemented monocytes prior to overnight storage (day 0)

| Donor # | Volume (mL) | Cell concentration/mL | Total Nucleated Cells | % viability | % Viable monocytes (FACs) |
| --- | --- | --- | --- | --- | --- |
| 1 | 202 | 8.47E+06 | 1.71E+09 | 99 | 83.7 |
| 2 | 201 | 7.51E+06 | 1.51E+09 | 99 | 76.3 |
| 3 | 210 | 7.41E+06 | 1.56E+09 | 97 | 67.0 |
| 4 | 298 | 1.03E+07 | 3.07E+09 | 99 | 75.1 |

S4. Stored bag cell viability and purity (day 1)

| Donor # | Volume (mL) | Cell concentration/mL | Total Nucleated Cells | % viability | % Viable monocytes (FACs) |
| --- | --- | --- | --- | --- | --- |
| 1 | 202 | 8.34E+06 | 1.68E+09 | 97 | 83.1 |
| 2 | 201 | 7.17E+06 | 1.44E+09 | 99 | 81.5 |
| 3 | 209 | 6.90E+06 | 1.44E+09 | 98 | 65.6 |
| 4 | 296 | 1.00E+07 | 2.96E+09 | 95 | 74.8 |

S5. Cell recovery after overnight storage

| Donor # | Cell recovery |
| --- | --- |
| 1 | 98% |
| 2 | 95% |
| 3 | 92% |
| 4 | 96% |

S6. Final product safety assays

| Donor | FN bacterial sterility | FN fungal sterility | Mycoplasma PCR | Endotoxin |
| --- | --- | --- | --- | --- |
| 1 | No growth | No growth of fungus | Negative | NA |
| 2 | No growth | No growth of fungus | Negative | <5 EU/mL |
| 3 | No growth | No growth of fungus | Negative | <5 EU/mL |
| 4 | No growth | No growth of fungus | Negative | <5 EU/mL |

S7: Cryopreserved cell recovery

| Sample | TNC | | | CD14+/CD16+ | | |
| --- | --- | --- | --- | --- | --- | --- |
|  | Pre Thaw | Post Thaw | Recovery | Pre Thaw | Post Thaw | Recovery |
| 1 | 3.50E+08 | 3.50E+08 | 100.0% | 2.85E+08 | 2.94E+08 | 103.3% |
| 2 | 4.22E+08 | 3.97E+08 | 94.1% | 3.16E+08 | 3.12E+08 | 98.6% |
| 3 | 3.33E+08 | 2.93E+08 | 88.0% | 2.60E+08 | 2.37E+08 | 91.3% |

S8: Frequency of CD14/CD16 monocytes

| Sample | Fresh sample | | | Cryo sample | |
| --- | --- | --- | --- | --- | --- |
|  | Bag | SM | FN | Day 1 | FN |
| 1 | 15.30% | 81.40% | 79.10% | 84.10% | 87.60% |
| 2 | 14.00% | 74.80% | 79.10% | 78.40% | 74.80% |
| 3 | 43.50% | 78.10% | 76.80% | 81.00% | 80.60% |

S9: Absolute cell number and recovery of CD14/CD16 monocytes, day 1 through FN

| Sample | Fresh sample | | | Cryo sample | | |
| --- | --- | --- | --- | --- | --- | --- |
|  | Day 1 | FN | Recovery | Day 1 | FN | Recovery |
| 1 | 7.50E+07 | 7.55E+07 | 100.67% | 2.45E+08 | 2.25E+08 | 91.84% |
| 2 | 2.16E+08 | 2.20E+08 | 101.85% | 2.80E+08 | 2.43E+08 | 86.82% |
| 3 | 7.50E+07 | 7.03E+07 | 93.73 | 2.61E+08 | 2.36E+08 | 90.34% |

S10: Functional assay (% Killing)

| Sample | Fresh sample | Cryo sample |
| --- | --- | --- |
| 1 | 94.1% | 95.8% |
| 2 | 96.1% | 94.7% |
| 3 | 95.7% | 95.3% |

S11: Materials used in development of the final product
