# Supplementary figures and images for: Production of a cellular product consisting of monocytes stimulated with Sylatron® (Peginterferon alfa-2b) and Actimmune® (Interferon gamma-1b) for human use
Source: J Transl Med. 2019 Mar 14;17:82. doi: 10.1186/s12967-019-1822-6 (PMC6419352; doi:10.1186/s12967-019-1822-6)

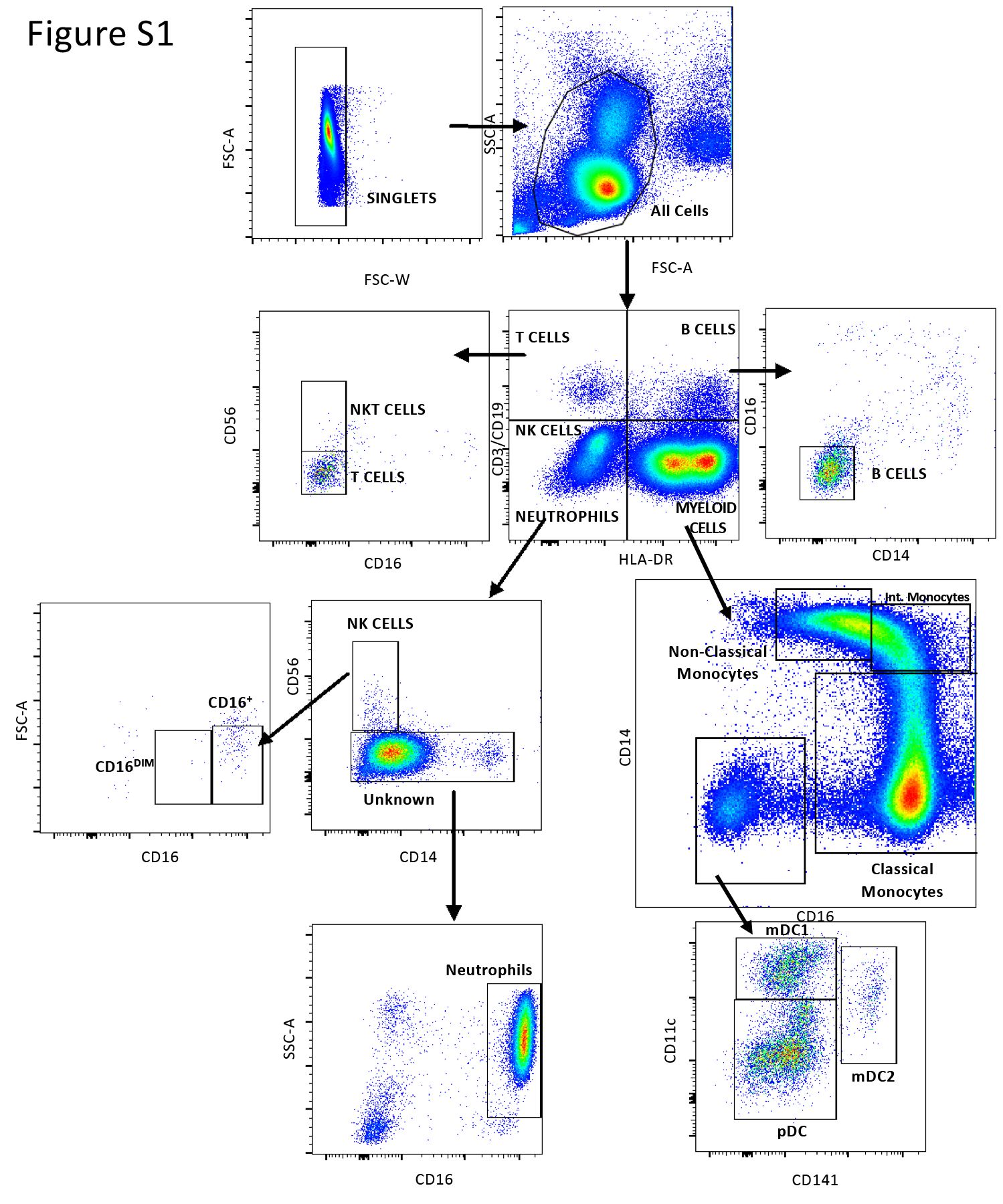

Supplement: Supplementary file 2 — Additional file 2: Figure S1. Expanded workflow of cells from counter flow elutriation showing flow cytometry results from RO fraction of Donor 1. [file 12967_2019_1822_MOESM2_ESM.tif]

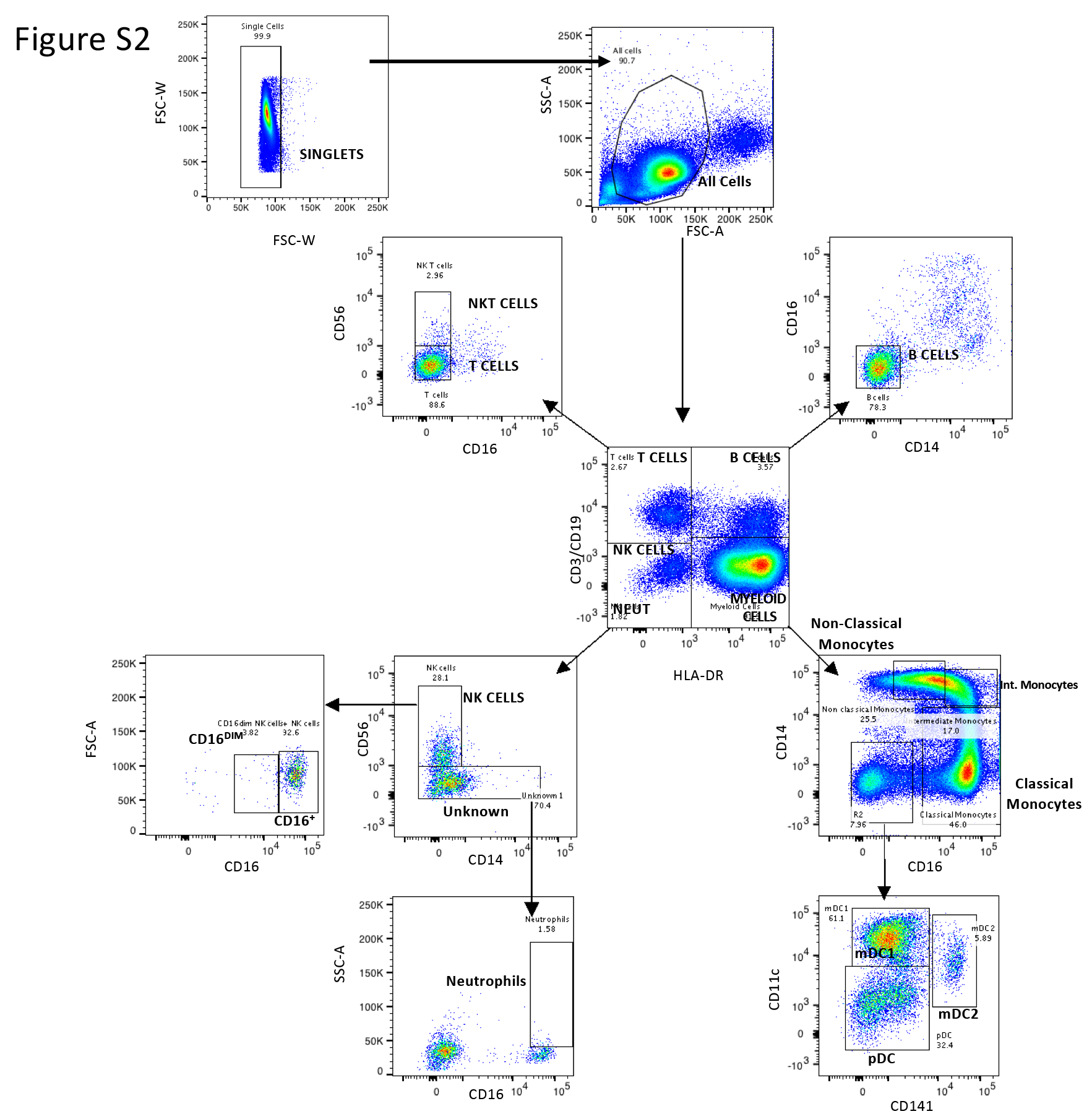

Supplement: Supplementary file 3 — Additional file 3: Figure S2. Expanded workflow of cells from counter flow elutriation showing flow cytometry results from 124 fraction of Donor 1. [file 12967_2019_1822_MOESM3_ESM.tif]

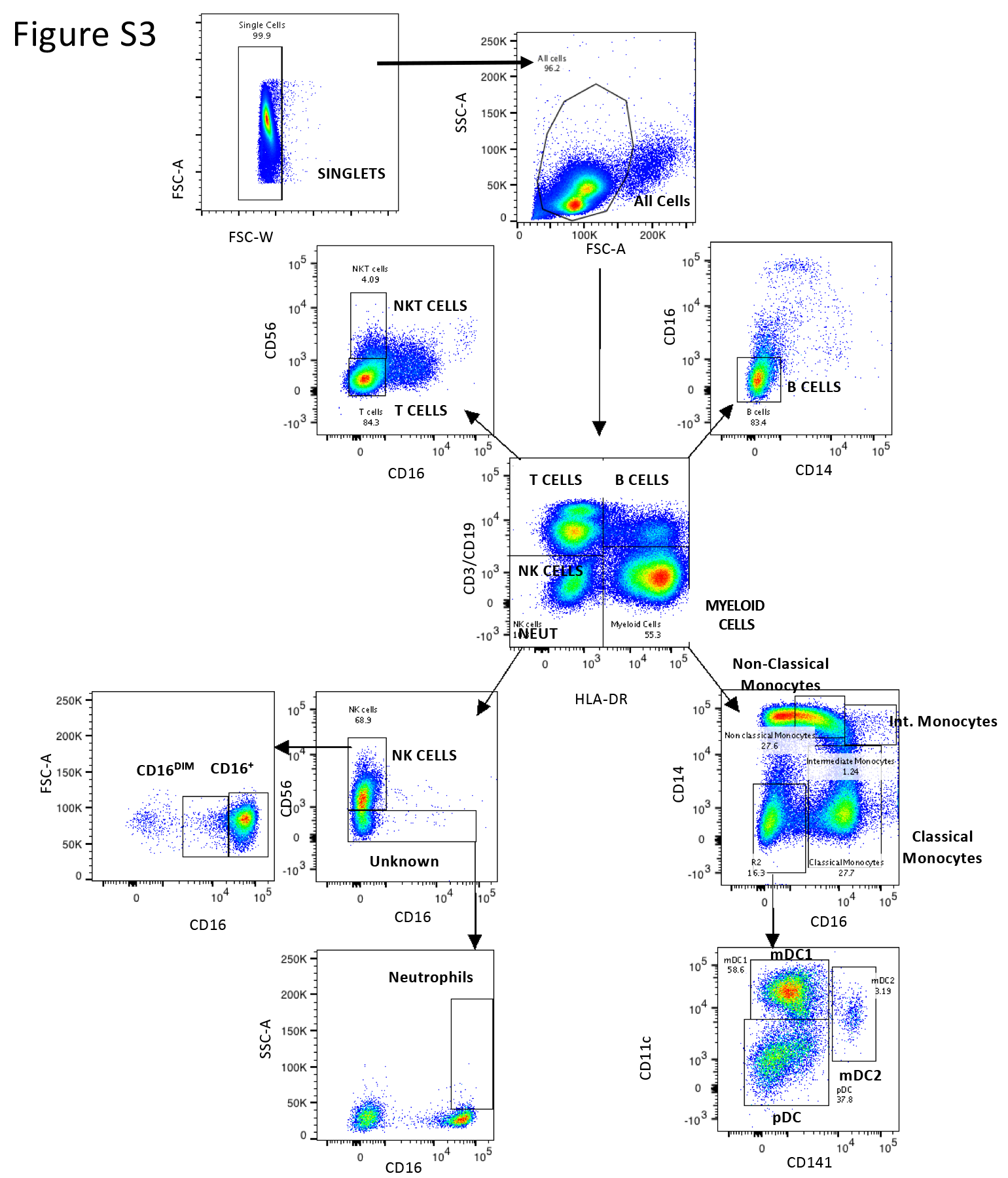

Supplement: Supplementary file 4 — Additional file 4: Figure S3. Expanded workflow of cells from counter flow elutriation showing flow cytometry results from 122 fraction of Donor 1. [file 12967_2019_1822_MOESM4_ESM.tif]

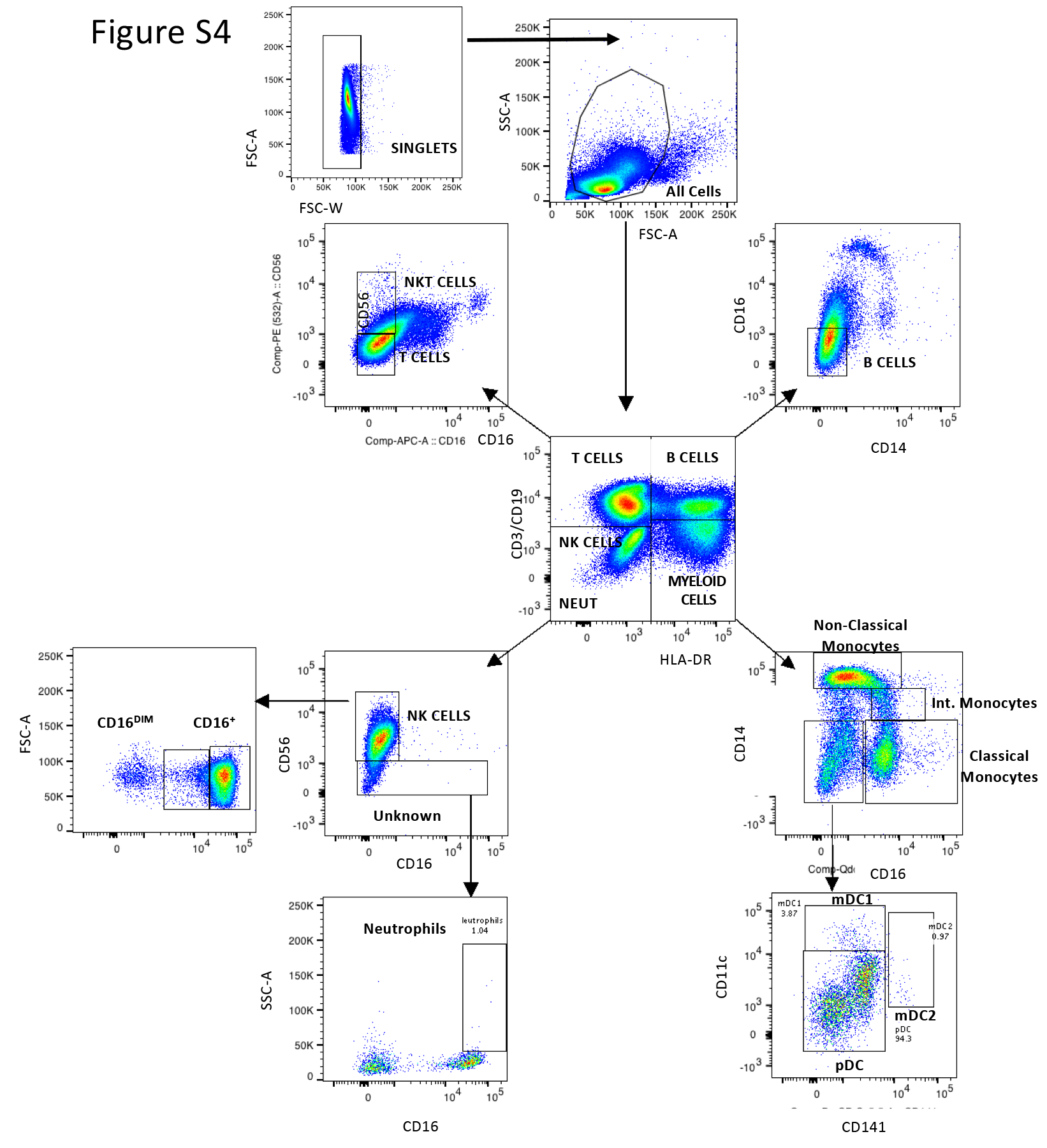

Supplement: Supplementary file 5 — Additional file 5: Figure S4. Expanded workflow of cells from counter flow elutriation showing flow cytometry results from 120 fraction of Donor 1. [file 12967_2019_1822_MOESM5_ESM.tif]
